# Supplementary material for: Towards an open and decentralized case law curation ecosystem
Source: PLoS One. 2020 Oct 2;15(10):e0240041. doi: 10.1371/journal.pone.0240041 (PMC7531852; doi:10.1371/journal.pone.0240041)
Supplement: Attachment — Submitted filename: rebuttal_letter.pdf [file pone.0240041.s001.pdf]

General response to the editor and the reviewers

We thank the editor and the reviewers for their critical assessment of our work. We agree with all the insightful comments and recommendations which lead us to the current re-submission. Next we address all issues raised in detail.

Editor

> Please ensure that your manuscript meets PLOS ONE's style requirements, including those for file naming. The PLOS ONE style templates can be found at
>
> `https://journals.plos.org/plosone/s/file?id=wjVg/PLOSOne_formatting_sample_main_body.pdf` and
>
> `https://journals.plos.org/plosone/s/file?id=ba62/PLOSOne_formatting_sample_title_authors_affiliations.pdf`

Reply: We have followed all requirements as those are found at the two links above.

> Please remove your figures from within your manuscript file, leaving only the individual TIFF/EPS image files, uploaded separately. These will be automatically included in the reviewers' PDF.

Reply: All figures (except those in latex) have been removed from the manuscript and uploaded separately as requested.

> Please upload a new copy of Figure 1 as the detail is not clear. Please follow the link for more information:
>
> `https://blogs.plos.org/plos/2019/06/looking-good-tips-for-creating-your-plos-figures-grap` [illegible]
>
> `https://blogs.plos.org/plos/2019/06/looking-good-tips-for-creating-your-plos-figures-grap` [illegible]

Reply: A new version of Figure 1 in latex that clearly reads all details has been uploaded.

> Please ensure that you refer to Figure 4 in your text as, if accepted, production will need this reference to link the reader to the figure.

Reply: We do refer to Figure 4, in the paragraph *Our overall ... these technologies interact.* found just before the beginning of section entitled "Synopsis and prospects". We have slightly modified this paragraph as well.

Reviewer 1

Reviewer Point P 1.1 — The study proposed a very interesting techno-legal which has potential to address the problem of centralized legal case repositories, and aid in the addressing some legal bottlenecks. However, the research content of the manuscript is largely ignored. For instance,

Reply: We fully agree that the research content of the manuscript is not apparent. We have modified the paper according to the points below and we believe that now the center of scientific gravity is closer to its research aspects.

Reviewer Point P 1.2 — The problem of unified format (”since no standard metadata structure has been agreed to represent a case law document”) for case law representation is largely ignored. This could have formed a major component of the research, upon which the development can be evaluated.

Reply: The reviewer is correct, both interoperability and standardization is required. Specifically, we are aware of the necessity of syntactic and semantic data interoperability.

Nevertheless, our paper places substantial emphasis on the careful design of the rules and incentives that define the protocol as well as the combination of technologies and components that establish the platform's entire solution stack.

The suggested metadata structure of a legal document used in our paper serves as a flexible base on which our proposed protocol can be built. Any additional research in that area would complement but not invalidate our work.

To make this clear to our readers the above paragraph has been added in section "Synopsis and Prospects".

Reviewer Point P 1.3 — in the related works section, the study identified important literature in decentralized platforms, but largely ignored legal platforms, either decentralized or centralized. This would have formed the basis of comparison in the discussion section, Again, no such comparison is provide in the discussion section.

Reply: To the best of our knowledge, our system is the only attempt towards a decentralized platform for case law. The reviewer is correct on asking for a comparison of our system to existing centralized ones. While there is no dedicated section in our paper that directly serves this purpose, we believe that the following points can adequately justify its absence:

- We make clear in Section "Introduction" that our effort is the only known decentralized case law platform.
- In Section "Introduction" we enumerate the vital drawbacks of centralized legal platforms while presenting the advantages of decentralization.
- We comment on hybrid centralized/decentralized systems in a newly added paragraph in Section "Synopsis and prospects".

Reviewer Point P 1.4 — Evaluation of the system is largely missing in the report. Evaluation in this case can leverage software engineering approach, as well as information system approach

(where usability, relevance, content reliability, legal limitation, as well as potential challenges). This is where significant result is expected to be generated to validate and justify the study.

Reply: Again the reviewer is absolutely correct. Evaluation of our system has been missing. For this:

- Gas cost simulation data in a form of a graph and a table have been added to a new subsection at the end of our section entitled "Implementation". The results are thoroughly discussed in the same subsection.
- The "Anonymization" subsection under "Implementation" has been renamed to "Legal limitations" and now discusses more legal issues that could arise during the operation of our platform.

It is our belief that the above add significant scientific merit to our paper and we thank the reviewer for his suggestion.

Reviewer Point P 1.5 — The section on the reliability of the decentralization is vaguely presented. The study further attempted to highlight the need for a semi-decentralized platform. This section is ambiguously presented, and it constitutes a major research concern.

Reply: Please refer to the paragraph added in Section "Synopsis and prospects" which further discusses hybrid centralized/decentralized systems.

Reviewer Point P 1.6 — One would expect to see a comparative analysis of some sort, given the wide array of decentralized platform, and the legal case work-house identified in the introductory section of the manuscript.

Reply: We believe that this point is addressed in our comments in P1.3 above. We are more than willing to reconsider if the reviewer could further explain this issue.

Reviewer 2

Reviewer Point P 2.1 — The manuscript idea is novice and interesting. The author(s) proposed a public and decentralized platform that allows the submission of court decisions in a decentralized database. However, some design decisions need to be supported and some points need clarification. For example, why authors used Ethereum not waves or bitcoin. How system performance and security is assessed? How the system is compared to other systems? Are there any quantitative data we can rely on for the system assessment?

Reply:

- ✓We have added two paragraphs in the beginning of the sub section entitled "Ethereum and other blockchain platforms" that justifies why we have selected Ethereum as our blockchain platform.
- ✓The performance issue has been addressed in our answer of point 1.4 above.
- ✓We agree that security assessment is lacking. A new "Security" subsection which adresses this issue has been added at the end of Section "Implementation".

- ✓The data issue has also been addressed in our answer of point 1.4.

Please note that during the revision of our paper we made additional minor changes which we believe add value to our paper.
